# Can a continuous quality improvement program create culturally safe emergency departments for Aboriginal people in Australia? A multiple baseline study

**DOI:** 10.1186/s12913-019-4049-6

**Published:** 2019-04-11

**Authors:** Thomas Gadsden, Gai Wilson, James Totterdell, John Willis, Ashima Gupta, Alwin Chong, Angela Clarke, Michelle Winters, Kym Donahue, Sonia Posenelli, Louise Maher, Jessica Stewart, Helen Gardiner, Erin Passmore, Aaron Cashmore, Andrew Milat

**Affiliations:** 1 NSW Ministry of Health, 73 Miller Street, North Sydney, NSW 2060 Australia; 2 School of Public Health and Community Medicine, UNSW, Sydney, NSW 2052 Australia; 3 Positive Futures Research Collaboration, Division of Health Sciences, University of South Australia, Adelaide, SA 5001 Australia; 4 NSW Department of Family and Community Services, 223-239 Liverpool Road, Ashfield, NSW 2131 Australia; 5 University of Melbourne, Parkville, VIC 3010 Australia; 6 St Vincent’s Health Australia, Level 5, 340 Albert Street, Melbourne, VIC 3002 Australia; 7 St Vincent’s Hospital Melbourne, 41 Victoria Parade, Fitzroy, VIC 3065 Australia

**Keywords:** Indigenous health, Aboriginal health, Cultural safety, Emergency department, Incomplete emergency department visit

## Abstract

**Background:**

Providing culturally safe health care can contribute to improved health among Aboriginal people. However, little is known about how to make hospitals culturally safe for Aboriginal people. This study assessed the impact of an emergency department (ED)-based continuous quality improvement program on: the accuracy of recording of Aboriginal status in ED information systems; incomplete ED visits among Aboriginal patients; and the cultural appropriateness of ED systems and environments.

**Methods:**

Between 2012 and 2014, the Aboriginal Identification in Hospitals Quality Improvement Program (AIHQIP) was implemented in eight EDs in NSW, Australia. A multiple baseline design and analysis of linked administrative data were used to assess program impact on the proportion of Aboriginal patients correctly identified as Aboriginal in ED information systems and incomplete ED visits in Aboriginal patients. Key informant interviews and document review were used to explore organisational changes.

**Results:**

In all EDs combined, the AIHQIP was not associated with a reduction in incomplete ED visits in Aboriginal people, nor did it influence the proportion of ED visits made by Aboriginal people that had an accurate recording of Aboriginal status. However, in two EDs it was associated with an increase in the trend of accurate recording of Aboriginality from baseline to the intervention period (odds ratio (OR) 1.31, *p* < 0.001 in ED 4 and OR 1.15, *p* = 0.020 in ED 5). In other words, the accuracy of recording of Aboriginality increased from 61.4 to 70% in ED 4 and from 72.6 to 73.9% in ED 5. If the program were not implemented, only a marginal increase would have occurred in ED 4 (from 61.4 to 64%) and, in ED 5, the accuracy of reporting would have decreased (from 72.6 to 71.1%). Organisational changes were achieved across EDs, including modifications to waiting areas and improved processes for identifying Aboriginal patients and managing incomplete visits.

**Conclusions:**

The AIHQIP did not have an overall effect on the accuracy of recording of Aboriginal status or on levels of incomplete ED visits in Aboriginal patients. However, important organisational changes were achieved. Further research investigating the effectiveness of interventions to improve Aboriginal cultural safety is warranted.

**Electronic supplementary material:**

The online version of this article (10.1186/s12913-019-4049-6) contains supplementary material, which is available to authorized users.

## Background

In Australia, Aboriginal people^1^ have poorer health than non-Aboriginal people [1]. Although improvements have been made in recent decades, Aboriginal people continue to experience higher rates of infant mortality, injury, non-communicable diseases, preventable illnesses and nutritional disorders than other Australians [2, 3]. Several social factors contribute to the gap in health outcomes between Aboriginal and non-Aboriginal people, such as higher levels of unemployment, social exclusion and experiences of racism and suboptimal use of health care among Aboriginal people [3–5]. Aboriginal people are more likely than non-Aboriginal people to delay seeking care until their illness is advanced and/or comorbid [6].

Aboriginal peoples’ engagement with health care is influenced by the physical accessibility, affordability and cultural safety of health service provision [7]. Culturally safe care is characterised by a genuine partnership between patient and health care provider in which: power is shared; the life experiences, views and beliefs – especially cultural beliefs – of the patient are respected; and Aboriginal histories and associated social impacts are acknowledged [8–11]. Some Aboriginal people feel culturally unsafe when using mainstream health services in Australia and are therefore reluctant to seek care in these settings [12]. Some factors that undermine the cultural safety of Aboriginal patients include: culturally insensitive health staff; institutionalised racism; a shortage of Aboriginal health workers; distrust of the health system among some Aboriginal patients; and unwelcoming waiting areas in key settings like hospital emergency departments (ED) [13, 14].

Aboriginal people are overrepresented among ED patients, relative to population size [15]. Additionally, EDs are the first point of contact with the health system for many Aboriginal people. EDs are therefore uniquely positioned to improve the health of this group [16]. However, EDs can feel unwelcoming and culturally unsafe for some Aboriginal people [13, 14]. Common aspects of the ED visit experience, like variable and sometimes long wait times and a lack of information about ED processes, contribute to a perceived sense of powerlessness or lack of control among some Aboriginal people [13]. Further, rates of incomplete ED visits (where the patient either left the ED before receiving a medical assessment or left the ED after a medical assessment but before completion of care) are elevated among Aboriginal people compared to non-Aboriginal people (9.7% vs. 7.1%), providing indirect evidence of higher levels of dissatisfaction with ED care in Aboriginal people than in other Australians [17–19].

Improving the cultural safety of health services can contribute to improved health among Aboriginal people and is a health priority in Australia [20, 21]. However, little is known about how to effectively intervene, including in ED settings. Recent reviews have found a predominance of descriptive studies, a lack of high quality intervention studies and a need for more rigorous innovation testing and translational research [10, 22, 23]. Continuous quality improvement (CQI) seeks to improve service quality through on-going cycles of reflection and refinement. Existing organisational processes are examined and modifications are developed, tested and, if effective, adopted [24]. CQI approaches have shown initial promise in improving the cultural safety of Aboriginal people in some primary care settings – especially in Aboriginal community-controlled health services – and warrant further investigation in secondary and tertiary care settings [10, 24].

We evaluated the Aboriginal Identification in Hospitals Quality Improvement Program (AIHQIP), a CQI initiative aiming to improve the cultural safety of Aboriginal patients in eight EDs in New South Wales (NSW), Australia. The study aimed to investigate whether the program: increased the proportion of Aboriginal patients correctly identified as Aboriginal in ED information systems; reduced the proportion of Aboriginal patients who had an incomplete ED visit; and improved the cultural appropriateness of ED systems and environments.

## Methods

### Intervention

Each participating ED implemented a CQI project with a focus on working with Aboriginal people to improve the cultural safety of ED services for Aboriginal patients. Each ED employed a project officer and established a working group to lead and guide project implementation. Working group membership typically included key hospital staff, staff of Aboriginal community-controlled organisations and local Aboriginal community members.

**Fig. 1:**
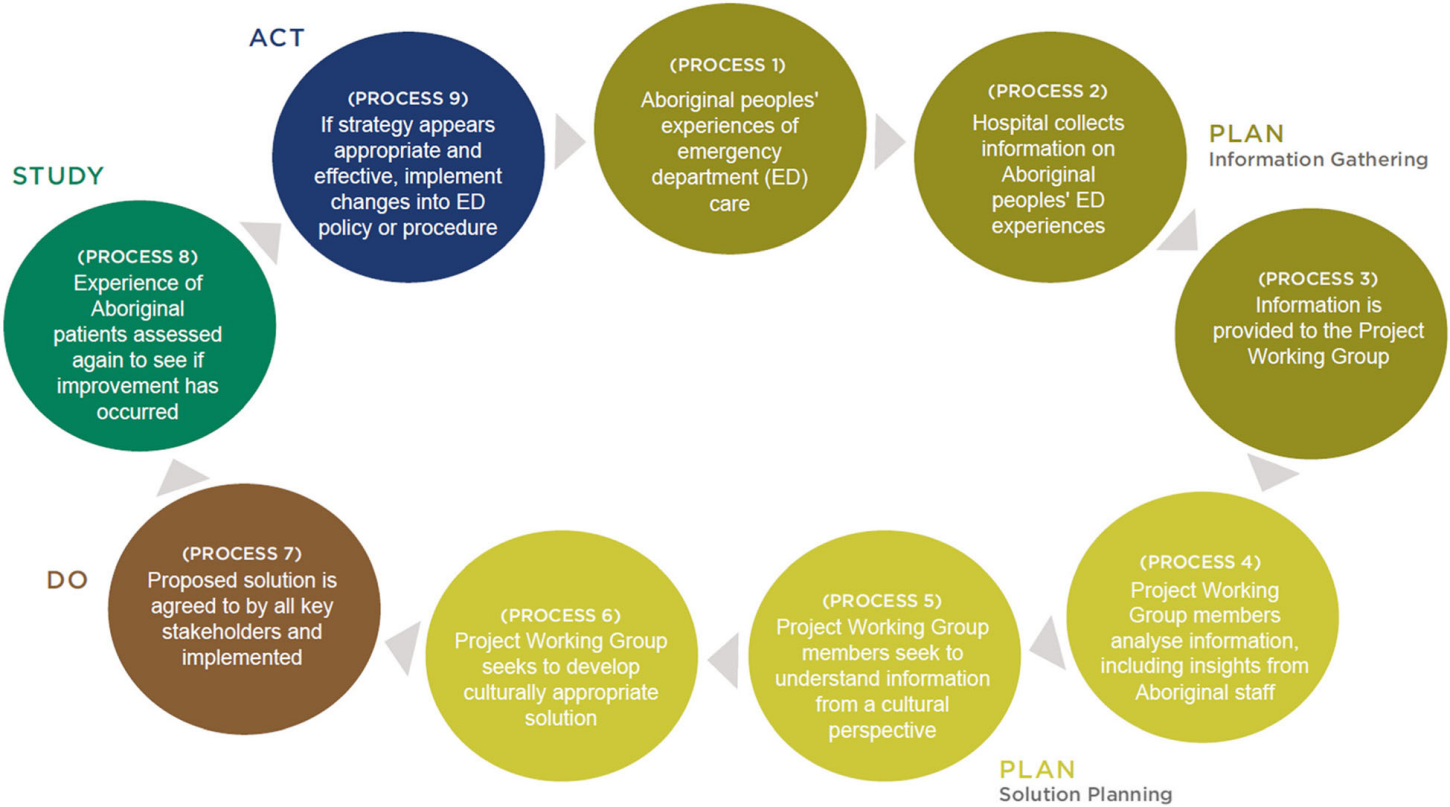
The Nine-step CQI framework used in the study. NA

The research team supported EDs to implement their CQI projects in the following ways:

1. Provision of a nine-step “Plan, Do, Study, Act” CQI framework with a unique emphasis on genuine partnership with Aboriginal communities. The framework included an implementation toolkit with supportive resources, such as an action plan template and examples of activities to aid each framework step. The nine steps of the framework are shown in Fig. 1, with further details available elsewhere [25].
2. Provision of a 1.5 day face-to-face training session for project officers and other interested working group members on how to implement the nine-step CQI framework and use the implementation toolkit. Key topics covered included: problem identification and solution generation; data collection and synthesis; action planning; guiding principles for collaborating with Aboriginal organisations; examples of best practice in the application of CQI; and the Aboriginal health policy context in NSW.
3. Provision of between four and six site visits during which project officers were provided with tailored CQI and cultural competency advice, mentoring and resources, with additional support provided via email and telephone.
4. Establishment of a network of project officers and other working group members from participating EDs to: share and discuss their CQI experiences; identify key facilitators and barriers to effective implementation; and share CQI resources. Two face-to-face meetings of the network were facilitated by the research team.

Each ED selected objectives for their CQI project from a predetermined list of objectives aligning with the overarching aims of the AIHQIP. Common project objectives included to: encourage Aboriginal patients to identify as Aboriginal in the ED; improve the Aboriginal cultural competence of ED staff and other hospital staff; improve collaboration between the ED and Aboriginal community-controlled organisations; and reduce incomplete ED visits among Aboriginal patients. Tailored strategies were developed, implemented and refined to meet these objectives.

### Setting

The AIHQIP was implemented in eight public EDs in NSW, one of eight States and Territories in Australia. About 7.5 million people live in NSW, of whom about 3% (n~ 216,000 persons) are Aboriginal people [26]. There are about 2.7 million visits to NSW EDs annually, with about 6% (n~ 462,000) of visits made by Aboriginal people [15].

In NSW, public EDs are operated by autonomous, geography-based health corporations called Local Health Districts, while the NSW Ministry of Health monitors and manages ED performance – for example, levels of re-presentation to the same ED within 48 h among Aboriginal patients. Several policies and procedures are in place to improve Aboriginal peoples’ experiences of emergency care in NSW, including mandatory Aboriginal cultural competency training for all hospital staff, mandatory recording of the Aboriginal status of hospital patients in information systems and a strategy for increasing and building the capacity of the Aboriginal health workforce in public hospitals, including EDs. Additionally, some EDs employ Aboriginal Liaison Officers (ALO) to provide emotional, social and cultural support to Aboriginal patients and their families when in hospital.

#### Study sites

Site selection was non-random. EDs were chosen to include a mix of rural and metropolitan EDs across multiple Local Health Districts. Additionally, executive support for implementing the AIHQIP and employment of an ALO were essential criterion for study participation, as these characteristics were considered fundamental to implementing the program as intended. The characteristics of participating EDs are included in Table 1.

**Table 1:** Characteristics of participating EDs^a, b^

Study ED	Number of ED visits		Location	Aboriginal Liaison Officer employed	% of Local Health District population who identified as Aboriginal people		Length of AIHQIP implementation
	2010	2015			2010	2015	
1	11,534^c^	25,016	Rural	Yes	11	11.6	10 months
2	15,990^c^	23,943	Rural	Yes	4.7	5	14 months
3	19,268	24,410	Rural	Yes	5.8	6.2	15 months
4	61,590	80,264	Metropolitan	Yes	1.8	1.8	15 months
5	46,355^c^	72,886	Metropolitan	Yes	1.1	1.1	12 months
6	25,631	29,548	Rural	Yes	3.3	3.4	12 months
7	41,306	47,825	Metropolitan	Yes	0.9	1	15 months
8	51,340	67,329	Metropolitan	Yes	3.1	3.2	10 months

^a^Sources: Centre for Epidemiology and Evidence. Health Statistics New South Wales. Sydney: NSW Ministry of Health. Available at: www.healthstats.nsw.gov.au. Accessed (07/11/17); Bureau of Health Information. Healthcare Observer. Sydney. Available at: www.bhi.nsw.gov.au/Healthcare_Observer/_nocache. Accessed (05/02/18)

^b^Study period: between 1st January 2010 and 31st March 2015

^c^Incomplete data in this calendar year.

A multiple baseline design, qualitative interviews with program stakeholders and document review were used to evaluate the AIHQIP.

### Multiple baseline design

A multiple baseline design [27] and secondary analysis of linked administrative health data were used to assess quantitative outcomes of the AIHQIP.

#### Data source

Data were obtained from the NSW Admitted Patient, Emergency Department Attendance and Deaths Register (APEDDR). The APEDDR is a statutory public health and disease register established under the *NSW Public Health Act 2010* to support, among other things, evaluations of public health interventions. It includes linked records of the following datasets:

- *NSW Admitted Patient Data Collection* includes records of all separations in NSW private and public hospitals.
- *NSW Register of Births, Deaths and Marriages* includes records of all births, deaths and marriages registered in NSW.
- *Cause of Death Unit Record File* provides cause(s) of death for all deaths registered in NSW.
- *NSW Emergency Department Data Collection* includes records of all visits to public EDs in NSW. Data are obtained from patient information systems in local EDs.

These data sources were linked by the Centre for Health Record Linkage using probabilistic record linkage methods.

#### Study population

The study population included Aboriginal people who attended one of the eight participating EDs between 1st January 2010 and 31st March 2015.

#### Outcomes

The two outcomes investigated were the proportion of Aboriginal patients correctly identified as Aboriginal in ED information systems and rates of incomplete ED visits. Both are considered indirect indicators of the cultural safety of ED service provision [23].

In NSW, staff in public EDs are required to ask every ED patient if they are Aboriginal^2^ although adherence to this requirement is variable within and across EDs. Health staff are more likely to ask this question of patients if they are culturally competent and work in an environment in which Aboriginal cultural competency standards are integrated into policies and procedures [28]. An Aboriginal person may choose not to identify as Aboriginal if they feel culturally unsafe in the ED, especially if they think that identifying as Aboriginal will negatively influence the quality of care they receive [29, 30].

The proportion of Aboriginal patients correctly identified as Aboriginal in ED information systems was assessed using the Enhanced Reporting of Aboriginality (ERA) variable in the APEDDR. The different datasets in the APEDDR and records of the *NSW Perinatal Data Collection*^3^ (all of which hold information on the Aboriginal status of the patient/person) were linked and an algorithm applied to identify ED records for which the patient could reasonably be considered an Aboriginal person for analysis purposes. The algorithm was as follows: if the person had three or more independent units of information (for example, a birth record, a hospital separation record and an ED separation record) and at least two of these indicated that the person was Aboriginal, then the person was considered Aboriginal; or if the person had one or two independent units of information and at least one of these indicated that the person was Aboriginal, then the person was considered Aboriginal. More on the ERA variable can be found elsewhere [31]. The accuracy of recording of Aboriginality was calculated as the observed number of ED visits for which the patient was recorded as Aboriginal in the ED information system divided by the “expected” number of ED visits by Aboriginal people, as determined by the ERA calculation.

Incomplete ED visits included visits for which the patient either left the ED before receiving a medical assessment or left the ED after a medical assessment but before completion of care or ED discharge. Feeling culturally unsafe is a main reason why Aboriginal people leave the ED early [14, 32]. Incomplete ED visits provide indirect evidence of patient dissatisfaction with the ED experience [19]. Rates of incomplete ED visits among Aboriginal people were calculated by dividing the number of incomplete visits by the total number of ED visits, using the Indigenous status variable in the NSW Emergency Department Data Collection.

#### Procedure

The eight EDs were allocated to three clusters. The clustering of EDs was non-random and informed by practical considerations, such as readiness to start implementing the AIHQIP. Clusters were then randomly assigned an implementation order of first, second or third. It was intended that program implementation in each cluster would be separated by three months. However, some EDs experienced delays in implementing the AIHQIP such that the period separating program commencement in EDs ranged from one month to four months. The first cluster of EDs started implementation in August 2012, with the implementation period at each site ranging in duration from 10 months to 15 months (Table 1).

Monthly data on the two outcomes were obtained for each ED and partitioned temporally into: before the intervention was implemented (control period: January 1st 2010 until the first site visit by the research team^d^^4^) and during and immediately after program implementation (intervention period: from the date of the first site visit until March 31st 2015). Each ED had at least 17 control period time points and 19 intervention period time points.

The multiple baseline design acknowledges that EDs differ by catchment population, staffing arrangements, administrative procedures and other characteristics. This variation, combined with the random staggering of intervention commencement across participating EDs (described above), increases the likelihood that any detected improvement in outcomes is due to the intervention, rather than external factors [27].

#### Statistical analysis

In determining program effectiveness, the main consideration was whether the trend in each outcome changed significantly, and in the desired direction, following program implementation. Change in intercept (that is, immediate change in the outcome at the start of the intervention period) was considered less relevant given the organisational changes the AIHQIP aimed to achieve can take a long time to establish.

Data were aggregated by month of ED presentation, hospital and patient sex, age group and Aboriginal status. Baseline patient characteristics were calculated for each site. Two modelling phases were undertaken for each outcome. First, preliminary logistic regression models were prepared for each study ED to investigate facility-level intervention effects. Following this, generalised linear mixed models were prepared to investigate the average intervention effect among all study EDs. Preliminary analyses identified a need to adjust for varying intercepts, trends and intervention effects between study sites. In all models, the key parameter of interest was the change in the linear trend from the control period to the intervention period. Parameter estimates were calculated, along with odds ratios, 95% confidence intervals and corresponding *p*-values. Data management and aggregations were performed in SAS version 9.3. Modelling was conducted in R version 3.2.1. An additional file provides a complete description of the statistical analysis [see Additional file 1].

### Qualitative interviews with program stakeholders and document review

Following implementation of the AIHQIP, qualitative interviews of 15–60 min duration were conducted with participating ED staff and other hospital staff (*n* = 23) exploring factors influencing project implementation and perceived achievements. Interviewees were AIHQIP project officers (*n* = 8), hospital managerial staff (n = 8), a hospital executive, an LHD Deputy Director of Aboriginal Health, and ALOs (*n* = 5). Six of the 23 interviewees were Aboriginal people.

Interviews were conducted in a quiet room in participating EDs (three were done by phone) by a non-Aboriginal researcher with experience conducting qualitative interviews with Aboriginal people. Detailed notes were taken during the interviews and circulated to interviewees for comment and verification. The notes were analysed using qualitative thematic analysis and a data mining approach. Responses were grouped under the questions that were asked during interviews. Following this, responses were repeatedly read to identify common themes and key achievements, both within individual EDs and across all sites.

Documents relating to the AIHQIP (*n* = 50) were also reviewed to describe changes to participating ED systems and environments relating to the cultural safety of Aboriginal patients. The main documents reviewed were site visit reports. Following each site visit, the research team and local working groups compiled a report using a standardised template. Reports described: the hospital context; the CQI support provided; implementation progress and challenges; and organisational changes perceived to be related to the AIHQIP. Documents were analysed using qualitative thematic analysis and a data mining approach (as described above), and findings triangulated with key informant interview findings. Several other methods were used as part of a comprehensive process evaluation of the AIHQIP. However, these are not the focus of this paper.

### Ethics approvals

Ethics approval was obtained from the NSW Population and Health Services Research Ethics Committee (Reference no. HREC/12/CIPHS/64) and the Aboriginal Health and Medical Research Council of NSW Ethics Committee (Reference no. 856/12).

## Results

During the control period, the average number of ED visits per month in the eight study EDs combined was 27,096 (Table 2). The average age of the presenting population was 28.3 years. However, average age varied among participating EDs (ranging from 25.2 years to 40.4 years). About half (51.3%) of the visits were made by females, although this also varied among participating EDs (ranging from 34.2 to 55.8%). Similarly, the proportion of ED presenters who were recorded as Aboriginal people in ED information systems differed between study sites, ranging from 1.7 to 22.3% (avg. 5.1%) (Table 2).

**Table 2:** Characteristics of patients visiting participating EDs in the control period^a^

Study EDs									
Control period characteristics (1st January 2010 to first site visit)	1	2	3	4	5	6	7	8	Total
Date of first site visit (month/year)	7/13	5/13	2/13	10/12	5/13	5/13	10/12	9/13	N/A
Months in control period	42	40	37	33	40	40	33	17	N/A
Average number of visits per month	1991	1885	1747	5299	5525	2211	3496	4942	27,096
Presentations by Aboriginal people									
Average number per month	132	167	389	104	176	91	60	227	1346
% of all ED presentations	6.6	8.9	22.3	2.0	3.2	4.1	1.7	4.6	5.1
Accuracy of recording of Aboriginality (%)	77.6	79.1	87.2	61.4	72.6	77.4	45.5	79.6	76.0
Incomplete visits in Aboriginal people (%)	7.6	9.6	6.5	16.9	14.8	20.6	24.9	9.6	11.5
Sex									
Male (%)	45.2	50.3	48.7	44.2	46.4	52.5	65.8	46.9	48.7
Female (%)	54.8	49.7	51.3	55.8	53.6	47.5	34.2	53.1	51.3
Age (years)									
0–14	31.5	34.6	35.7	21.9	13.9	29.3	0.2	25.0	27.8
15–29	33.3	31.3	27.1	33.4	26.4	30.1	22.8	33.9	29.4
30–44	18.1	17.2	19.4	20.0	25.5	21.0	42.7	19.9	21.0
45–59	12.0	10.8	13.2	14.6	22.4	13.7	27.9	13.8	14.9
60–74	4.7	4.6	4.1	7.6	9.5	4.6	5.3	6.1	5.6
75+	0.4	1.5	0.5	2.6	2.3	1.4	1.3	1.2	1.2
Average (SD)	25.4 (18.3)	25.3 (19.2)	25.2 (19.1)	30.5 (20.6)	36.2 (19.7)	27.4 (19.0)	40.4 (13.0)	28.3 (19.3)	28.3 (19.6)

^a^Source: Admitted Patient, Emergency Department Attendance and Deaths Register, NSW Ministry of Health SAPHaRI

During the control period, the proportion of ED visits made by Aboriginal people that had an accurate recording of Aboriginal status varied among participating EDs (ranging from 45.5 to 87.2%; avg. 76%). The proportion of ED visits made by Aboriginal people that were incomplete also differed between study sites (ranging from 6.5 to 24.9%; avg. 11.5%) (Table 2).

### Recording of Aboriginal status in ED information systems

**Fig. 2:**
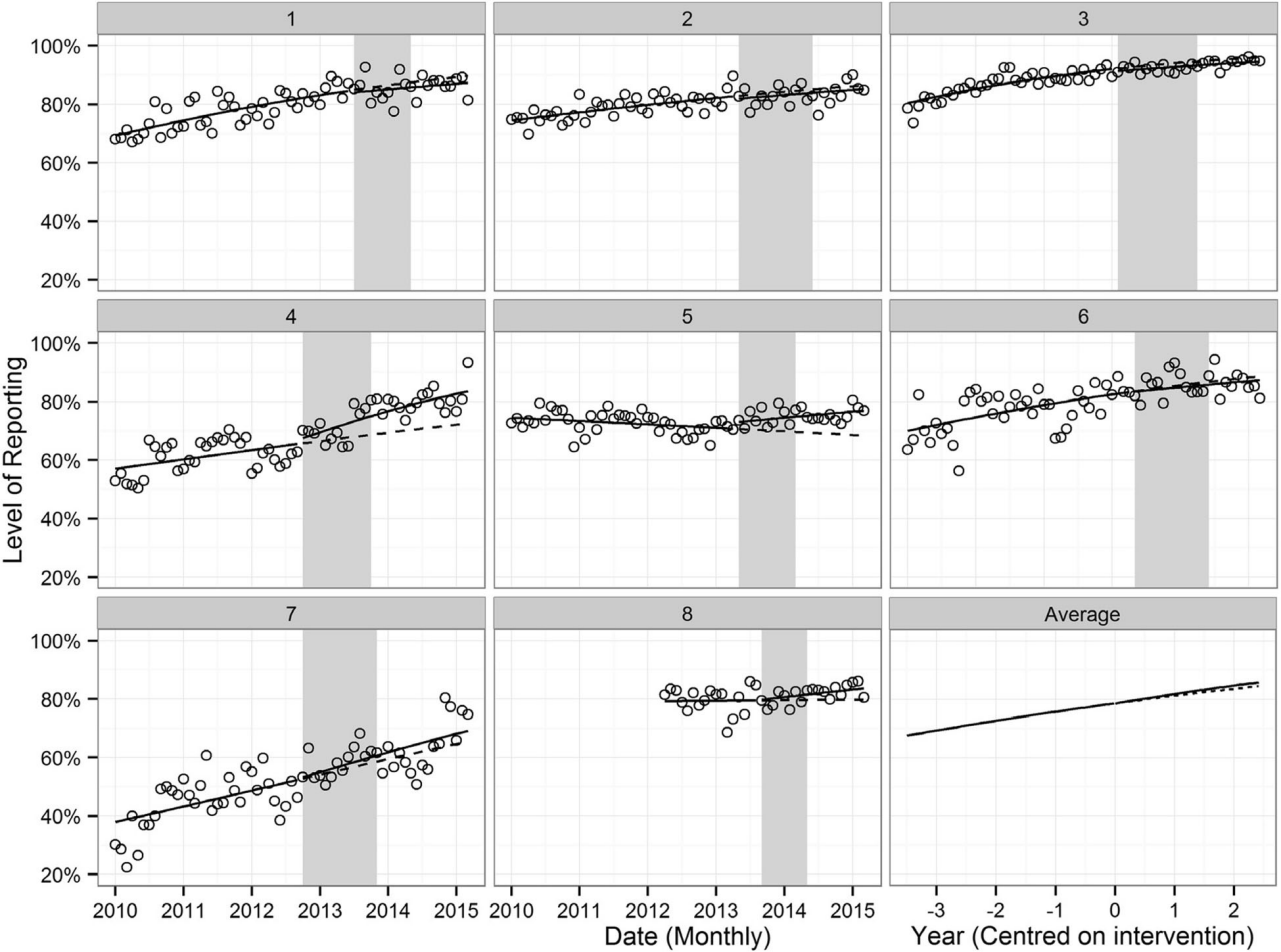
The proportion of ED visits made by Aboriginal people that had an accurate recording of Aboriginal status for eight study EDs^1, 2^ The shaded region indicates the intervention period for each hospital (first site visit to last site visit). Solid lines model the observed level of recording pre- and post-intervention. Dashed lines model the predicted level of recording had the intervention not occurred, based on pre-intervention trends. Source: Admitted Patient, Emergency Department Attendance and Deaths Register, NSW Ministry of Health SAPHaRI

During the control period, the proportion of ED visits made by Aboriginal people that had an accurate recording of Aboriginal status was significantly increasing over time in six of the eight participating EDs, while the accuracy of recording was decreasing significantly in one ED. A non-significant increase was observed in one ED (Fig. 2 and Table 3).

**Table 3:** Site-specific model estimates of the proportion of ED visits made by Aboriginal people that had an accurate recording of Aboriginal status^b^

Site-specific model estimates					
ED	Parameter	Estimate (log-odds)	Odds ratio	95% CI	P-value
1^a^					
	Pre-intervention slope	0.267	1.31	[1.21, 1.41]	< 0.001
	Change in slope	−0.197	0.82	[0.64, 1.06]	0.124
	Intervention slope	0.069	1.07	[0.84, 1.36]	0.572
	Change in intercept	0.004	1.00	[0.75, 1.34]	0.977
2					
	Pre-intervention slope	0.172	1.19	[1.12, 1.25]	< 0.001
	Change in slope	−0.001	1.00	[0.86, 1.16]	0.992
	Intervention slope	0.171	1.19	[1.04, 1.36]	0.013
	Change in intercept	−0.175	0.84	[0.70, 1.00]	0.054
3^a^					
	Pre-intervention slope	0.346	1.41	[1.31, 1.52]	< 0.001
	Change in slope	−0.080	0.92	[0.78, 1.09]	0.353
	Intervention slope	0.266	1.30	[1.12, 1.52]	< 0.001
	Change in intercept	−0.137	0.87	[0.70, 1.09]	0.231
4^a^					
	Pre-intervention slope	0.112	1.12	[1.01, 1.24]	0.028
	Change in slope	**0.273**	**1.31**	**[1.12, 1.54]**	**< 0.001**
	Intervention slope	0.385	1.47	[1.30, 1.66]	< 0.001
	Change in intercept	0.115	1.12	[0.90, 1.41]	0.316
5					
	Pre-intervention slope	−0.070	0.93	[0.89, 0.98]	0.003
	Change in slope	**0.139**	**1.15**	**[1.02, 1.29]**	**0.020**
	Intervention slope	0.068	1.07	[0.96, 1.19]	0.210
	Change in intercept	0.191	1.21	[1.05, 1.40]	0.009
6^a^					
	Pre-intervention slope	0.219	1.25	[1.12, 1.39]	< 0.001
	Change in slope	−0.209	0.81	[0.60, 1.10]	0.179
	Intervention slope	0.011	1.01	[0.76, 1.34]	0.940
	Change in intercept	0.208	1.23	[0.85, 1.79]	0.274
7^a^					
	Pre-intervention slope	0.244	1.28	[1.12, 1.46]	< 0.001
	Change in slope	0.042	1.04	[0.86, 1.26]	0.658
	Intervention slope	0.286	1.33	[1.17, 1.52]	< 0.001
	Change in intercept	− 0.033	0.97	[0.73, 1.27]	0.816
8^a^					
	Pre-intervention slope	−0.054	0.95	[0.72, 1.25]	0.701
	Change in slope	0.302	1.35	[0.95, 1.92]	0.093
	Intervention slope	0.248	1.28	[1.02, 1.60]	0.029
	Change in intercept	−0.024	0.98	[0.73, 1.31]	0.875

^a^Overdispersion accounted for by including dispersion parameter in analysis

^b^Source: Admitted Patient, Emergency Department Attendance and Deaths Register, NSW Ministry of Health SAPHaRI

The bold figures indicate a statiscally significant increase in the trend of accurate recording of Aboriginality from baseline to the intervention period

In two EDs, the AIHQIP was associated with a statistically significant increase in the trend of accurate recording of Aboriginality from the control period to the intervention period (Table 3) (the key parameter to consider in the table is the change in slope).

In ED 4, during the control period the odds of an ED visit made by an Aboriginal person having an accurate recording of Aboriginal status were increasing by a factor of 1.12 per year. During the intervention period, the odds were increasing by a factor of 1.47 per year (*p* < 0.001). In other words, if the AIHQIP was not implemented the accuracy of recording would have increased from 61.4% in the control period to 64% 12 months after the program was first implemented. However, program implementation was associated with a larger increase in the accuracy of recording during this period – from 61.4 to 70%.

In ED 5, during the control period the odds of an ED visit made by an Aboriginal person having an accurate recording of Aboriginal status were decreasing by a factor of 0.93 per year. During the intervention period, the odds were increasing by a factor of 1.07 per year (*p* = 0.020) (Table 3). In other words, if the AIHQIP was not implemented the accuracy of recording would have decreased from 72.6% in the control period to 71.1% 12 months after the program was first implemented. However, program implementation was associated with an increase in the accuracy of recording during this period – from 72.6 to 73.9%.

In all EDs combined, the AIHQIP was not associated with a change in the trend of accurate recording of Aboriginality from the control period to the intervention period (Table 4).

**Table 4:** Multi-site model estimate of the proportion of ED visits made by Aboriginal people that had an accurate recording of Aboriginal status^a^

Parameter	Estimate (log-odds)	Odds ratio	95% CI	P-value
Pre-intervention slope	0.162	1.18	[1.07, 1.29]	< 0.001
Change in slope	0.044	1.05	[0.94, 1.17]	0.429
Intervention slope	0.206	1.23	[1.14, 1.33]	< 0.001
Change in intercept	−0.004	1.00	[0.91, 1.09]	0.925

^a^Source: Admitted Patient, Emergency Department Attendance and Deaths Register, NSW Ministry of Health SAPHaRI

### Incomplete ED visits

**Fig. 3:**
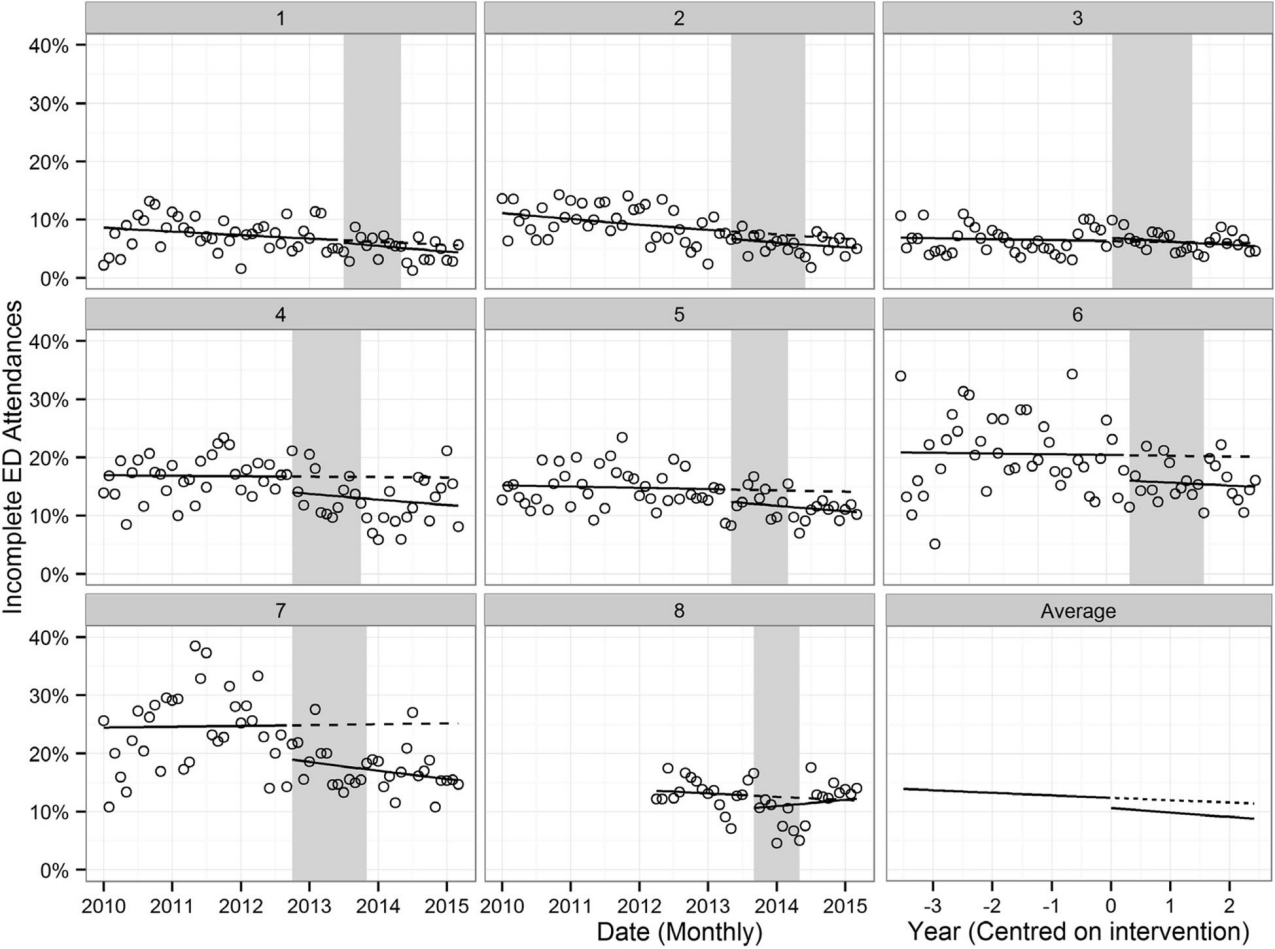
The proportion of ED visits made by Aboriginal people that were incomplete for eight participating EDs^1, 2^. The shaded region indicates the intervention delivery period for each hospital (first site visit to last site visit). Solid lines model the observed level of incomplete visits pre- and post-intervention. Dashed lines model the predicted level of incomplete visits if the intervention did not occur, based on pre-intervention trends. Source: Admitted Patient, Emergency Department Attendance and Deaths Register, NSW Ministry of Health SAPHaRI

During the control period, the proportion of ED visits made by Aboriginal people that were incomplete remained steady over time in seven of the eight participating EDs and decreased significantly in one ED (Fig. 3 and Table 5).

**Table 5:** Site-specific model estimates of incomplete ED visits in Aboriginal people^b^

Site specific model estimates					
ED	Parameter	Estimate (log-odds)	Odds ratio	95% CI	P-value
1					
	Pre-intervention slope	−0.049	0.95	[0.86, 1.05]	0.329
	Change in slope	−0.167	0.85	[0.60, 1.19]	0.331
	Intervention slope	−0.217	0.81	[0.58, 1.11]	0.188
	Change in intercept	−0.206	0.81	[0.56, 1.18]	0.272
2					
	Pre-intervention slope	−0.142	0.87	[0.80, 0.94]	0.001
	Change in slope	−0.011	0.99	[0.77, 1.27]	0.931
	Intervention slope	−0.153	0.86	[0.68, 1.09]	0.206
	Change in intercept	−0.183	0.83	[0.62, 1.12]	0.232
3^a^					
	Pre-intervention slope	−0.027	0.97	[0.86, 1.11]	0.681
	Change in slope	−0.119	0.89	[0.70, 1.12]	0.319
	Intervention slope	−0.145	0.86	[0.71, 1.05]	0.146
	Change in intercept	0.152	1.16	[0.84, 1.60]	0.354
4^a^					
	Pre-intervention slope	0.065	1.07	[0.92, 1.24]	0.402
	Change in slope	−0.144	0.87	[0.70, 1.08]	0.192
	Intervention slope	−0.080	0.92	[0.79, 1.08]	0.316
	Change in intercept	−0.314	0.73	[0.53, 1.00]	0.053
5					
	Pre-intervention slope	−0.019	0.98	[0.92, 1.05]	0.583
	Change in slope	−0.105	0.90	[0.75, 1.08]	0.254
	Intervention slope	−0.124	0.88	[0.75, 1.04]	0.144
	Change in intercept	−0.144	0.87	[0.70, 1.08]	0.196
6^a^					
	Pre-intervention slope	−0.030	0.97	[0.87, 1.09]	0.611
	Change in slope	−0.042	0.96	[0.72, 1.28]	0.779
	Intervention slope	−0.071	0.93	[0.71, 1.22]	0.601
	Change in intercept	−0.224	0.80	[0.56, 1.14]	0.216
7					
	Pre-intervention slope	0.024	1.02	[0.89, 1.17]	0.736
	Change in slope	−0.149	0.86	[0.72, 1.04]	0.119
	Intervention slope	−0.125	0.88	[0.78, 1.00]	0.052
	Change in intercept	−0.353	0.70	[0.54, 0.92]	0.011
8^a^					
	Pre-intervention slope	−0.154	0.86	[0.60, 1.23]	0.401
	Change in slope	0.431	1.54	[0.96, 2.46]	0.072
	Intervention slope	0.277	1.32	[0.98, 1.78]	0.072
	Change in intercept	−0.264	0.77	[0.51, 1.16]	0.213

^a^Overdispersion accounted for by including dispersion parameter in analysis

^b^Source: Admitted Patient, Emergency Department Attendance and Deaths Register, NSW Ministry of Health SAPHaRI

The AIHQIP was not associated with a decrease in the trend of incomplete ED visits among Aboriginal people from the control period to the intervention period in any of the eight study EDs (Table 5), nor was an overall program effect detected (Table 6). Although decreases in the trend were observed in some sites, these were not statistically significant (Table 5).

**Table 6:** Multi-site model estimate of incomplete ED visits in Aboriginal people^a^

Parameter	Estimate (cloglog)	95% CI	P-value
Pre-intervention slope	−0.036	[− 0.091, 0.019]	0.111
Change in slope	−0.047	[−0.175, 0.082]	0.378
Intervention slope	−0.083	[−0.199, 0.033]	0.083
Change in intercept	−0.161	[−0.307, − 0.014]	0.008

^a^Source: Admitted Patient, Emergency Department Attendance and Deaths Register, NSW Ministry of Health SAPHaRI

### Organisational changes

Table 7 provides an overview of CQI project objectives and examples of organisational changes achieved by study EDs. Some examples of organisational changes which key informants attributed to the AIHQIP include:

- the establishment of mandatory Aboriginal cultural competence training, including an Aboriginal patient identification training program, for ED staff and other hospital staff;
- the strengthening of existing referral mechanisms between the ED and local Aboriginal community-controlled health services;
- the establishment of a critical incident response procedure for all incomplete ED visits among Aboriginal patients, which aimed to learn from the incident, prevent similar incidents in the future and ensure follow up and care of the affected patient; and
- the establishment of a performance indicator dashboard on the ED care of Aboriginal patients, which aimed to monitor and guide ED and hospital practice.

**Table 7:** Overview of CQI project objectives and examples of organisational changes^a^

Project objectives	EDs targeting objective	Examples of observed organisational changes
Encourage Aboriginal patients to identify as Aboriginal in emergency departments (ED)	1, 2, 4, 5, 6, 7, 8	• Pamphlets and posters encouraging Aboriginal patients to identify as Aboriginal were established in EDs (n = 5) ^b^. • Training on how to ask patients about their Aboriginal status was delivered to ED staff and other hospital staff and embedded in staff orientation. Further, DVDs and other resources were developed to support this training (*n* = 6).
Maximise the quality of ED data and the use of these data to improve ED care for Aboriginal people	1, 4, 5, 7, 8	• Aboriginality was made a mandatory field in ED information systems and/or included in the patient registration screen (*n* = 3). • Fields for Aboriginal identification and incomplete ED visits in Aboriginal patients were included in the data query applications of ED information systems to facilitate routine reporting (n = 2). • A performance indicator dashboard on the ED care of Aboriginal patients was established to monitor and guide hospital practice (*n* = 1).
Increase the presence of Aboriginal Liaison Officers (ALO) in EDs	1, 2, 3, 4, 5, 6, 8	• Alert systems in ED information systems were established to link ALOs with Aboriginal patients (n = 1). • A practice guideline was established for ALOs to follow-up Aboriginal patients who have an incomplete ED visit (*n* = 1). • Messages about the availability of ALOs and how to use this service were streamed on televisions in the ED (n = 3).
Make ED and hospital wait areas welcoming for Aboriginal patients	1, 2, 4, 6, 7, 8	• Plaques acknowledging the traditional custodians of the land, maps describing the locations of Aboriginal clans and Aboriginal art were erected in ED wait areas (n = 5). • The Aboriginal “dreaming garden” in the hospital was redeveloped in partnership with the local Aboriginal Land Council (n = 1). • Survey of Aboriginal and other patients conducted and informed business case for redevelopment of ED wait area (n = 1).
Improve the Aboriginal cultural competence of ED staff and other hospital staff	1–8	• Aboriginal health workers (and sometimes non-Aboriginal staff) provided orientation to ED staff on Aboriginal cultural competence, local Aboriginal history and the roles of Aboriginal health workers and ALOs in the ED and hospital (n = 8). • Aboriginal cultural competence training was mandated and embedded in hospital staff orientation and training calendars (*n* = 8). • Hospital staff meetings implemented acknowledging events of significance to Aboriginal people (e.g. NAIDOC week) (n = 1)
Support the Aboriginal health workforce	1, 7	• A hospital Aboriginal employment strategy was established, which includes establishing an Aboriginal staff network, more Aboriginal-identified positions and mentoring programs for Aboriginal staff (n = 2).
Improve collaboration between the ED and Aboriginal community-controlled organisations	1–8	• A formal partnership agreement was established between the ED/hospital and the local Aboriginal community-controlled health service, which emphasised joint planning and service delivery and included provision for staff exchanges (n = 3). • Existing referral mechanisms between the ED and local Aboriginal community-controlled health services were refined (n = 1).
Reduce incomplete ED visits among Aboriginal patients	1–8	• An ED critical incident response procedure was established for incomplete visits in Aboriginal patients, which sought to learn from the incident, prevent similar incidents in the future and ensure follow up and care of the affected patient (n = 1). • Leaflets and electronic messaging were implemented in EDs explaining triage and administrative processes (n = 4). • A process for informing patients of wait times was embedded into routine practice, such as the shift handover procedure (*n* = 4).

^a^Similar project objectives have been combined. Findings come from key informant interviews and review of program documentation.

^b^ n indicates the number of EDs achieving this outcome.

Key informant interviewees identified several factors that they felt enabled program implementation, such as supportive hospital executive staff and a high level of engagement of local Aboriginal community-controlled health services and ALOs in project design and implementation. A more complete description of the factors influencing implementation is included in Additional file 2.

## Discussion

To our knowledge, this is the first study to comprehensively investigate the effectiveness of a CQI program in making EDs more culturally safe for Aboriginal people in Australia. Our complementary use of a quasi-experimental design investigating primary outcomes and qualitative methods exploring organisational changes and program implementation is particularly novel in this area. In all study EDs combined, the AIHQIP was not associated with a reduction in incomplete ED visits in Aboriginal people, nor did it influence the proportion of ED visits made by Aboriginal people that had an accurate recording of Aboriginal status. However, in two EDs the AIHQIP was associated with an increase in the trend of accurate recording of Aboriginality from the control period to the intervention period.

There are four main factors that might explain this limited evidence of an intervention effect. First, the intervention may have lacked the intensity to influence the primary outcomes investigated. Second, in some participating EDs recording of Aboriginal patients’ Aboriginality was fairly accurate and trending up at baseline. As such, improvements in this outcome may not have been detected, or indeed possible, in these EDs. The same issue applied to the incomplete ED visits outcome. Third, it is possible that the outcomes of interest were assessed prematurely, as the system changes that the AIHQIP sought to achieve can take considerable time to institutionalise [21]. Premature outcome assessment can occur in programs for Aboriginal people, especially if implementation is in complex systems where cause and effect and action and outcome relationships are poorly understood [33]. A longer intervention period may have increased the statistical power to detect intervention effects and provided more time for the program to produce change in the primary outcomes. This was not possible, however, due to constraints of time and resources. Similar multiple baseline studies might employ a longer intervention period. Lastly, we did not conduct power calculations prior to study implementation. Consequently, our study may have been under-powered to detect subtle changes associated with the intervention. That said, we analysed all ED records that were reasonably available to us during the five year study period.

The linked administrative health data used in this study have several strengths. They provide population-level insights, they are easy and inexpensive to obtain relative to primary data collection methods, and they draw on multiple health datasets and the ERA algorithm to provide as accurate a description of the study population as possible. However, the two outcomes used – Aboriginal patients correctly identified as Aboriginal in patient information systems and incomplete ED visits in Aboriginal people – may not be the most sensitive measures of culturally safe care. Complementary use of other quantitative methods, such as patient satisfaction surveys, may have provided a more sensitive and comprehensive way of measuring the impacts of the AIHQIP [34, 35]. As part of a comprehensive program of surveys, the Bureau of Health Information implements regular surveys of patients’ experiences of public hospital and emergency care in NSW, with some surveys oversampling Aboriginal patients [36]. These surveys provide a powerful tool for monitoring and evaluating the extent to which health services are meeting the needs of Aboriginal patients.

Our finding that the AIHQIP reoriented ED environments and systems towards the provision of culturally safe care for Aboriginal patients is encouraging. Organisations with cultural competency standards integrated into policies and practices have been found to foster culturally competent behaviours among health professionals [37]. It is therefore likely that the organisational changes achieved in this study, if sustained, will reinforce culturally safe care in participating EDs in the future.

A strength of our study was that the AIHQIP aligned with the available evidence on building cultural competency in health organisations. Although few studies have rigorously examined the effectiveness of cultural competency interventions, there is growing recognition of the need for sustained, multifaceted organisational-level approaches [10, 38]. Interventions that combine organisational change strategies with efforts to develop individual competency are more likely to be effective than stand-alone low intensity interventions [39, 40]. Similarly, the strong engagement of Aboriginal communities and staff in our study reflect best practice principles for working with Aboriginal people to improve health outcomes [20]. Our study reinforces the utility of CQI approaches as a tool for engaging Aboriginal communities in system change interventions. CQI should therefore form the basis of policy responses in this area.

### Limitations

There are three main limitations of our study. First, two participating EDs were delayed in commencing their CQI projects and therefore had condensed implementation periods, which may have reduced the likelihood of achieving program objectives and influencing the study outcomes. Second, the routinely collected data used in the multiple baseline design have some limitations relating to quality and completeness. In public EDs in NSW, practices relating to the entry of patient information in ED information systems, such as timeliness and quality checking, may vary among staff within EDs as well as between EDs, which introduces the potential of information bias in the findings. Third, the researcher who conducted the key informant interviews also conducted site visits in some participating EDs. This may have influenced some interviewees’ willingness to provide honest and fulsome responses during interviews.

## Conclusions

The AIHQIP did not have an overall effect on the accuracy of recording of Aboriginality in ED information systems or on levels of incomplete ED visits in Aboriginal patients. Still, key organisational changes were achieved, which over time may lead to more welcoming and culturally safe ED environments for Aboriginal patients. Further research investigating the effectiveness of interventions to improve Aboriginal cultural safety is warranted.

## Additional files

Additional file 1: Statistical analysis. A complete description of the statistical analysis used for the multiple baseline design. (DOCX 18 kb)

Additional file 2: Factors influencing implementation. Description of data: A detailed description of the factors influencing implementation, as reported by key informant interviewees. (DOCX 19 kb)

## Abbreviations

AIHQIP: Aboriginal Identification in Hospitals Quality Improvement Program
ALO: Aboriginal Liaison Officer
APEDDR: Admitted Patient, Emergency Department Attendance and Deaths Register
CQI: Continuous Quality Improvement
ED: Emergency Department
ERA: Enhanced Reporting of Aboriginality
NSW: New South Wales

## Footnotes

1 In this paper, Aboriginal and Torres Strait Islander people are referred to as Aboriginal people in recognition that Aboriginal people are the original inhabitants of the Australian state of NSW, where this study was conducted.

2 NSW Health clinical staff are required to ask the following question of all patients: *“Are you* (is the person) *of Aboriginal or Torres Strait Islander origin?”*

3 The *NSW Perinatal Data Collection* holds records of all births in NSW, regardless of the birth setting.

4 Due to data quality problems, the control period for ED 8 was from April 1st 2012 until the first site visit by the research team (September 1st 2013).
