# Supplementary material for: Can a continuous quality improvement program create culturally safe emergency departments for Aboriginal people in Australia? A multiple baseline study
Source: BMC Health Serv Res. 2019 Apr 11;19:222. doi: 10.1186/s12913-019-4049-6 (PMC6458761; doi:10.1186/s12913-019-4049-6)
Supplement: Supplementary file 1 — Statistical analysis. A complete description of the statistical analysis used for the multiple baseline design. (DOCX 18 kb) [file 12913_2019_4049_MOESM1_ESM.docx]

**ADDITIONAL FILE 1**

***Statistical analysis***

Summaries of patient demographic characteristics for each site were calculated and the outcomes were investigated with respect to sex and age groups. Two modelling phases were undertaken for each outcome. First, preliminary logistic regression models were prepared for each study emergency department (ED) to investigate facility-level intervention effects. Following this, a Generalized Linear Mixed Model (GLMM) was prepared to investigate the average intervention effect among all study EDs. Preliminary analyses identified a need to adjust for varying intercepts, trends and intervention effects between study sites. In all models, the key parameter of interest was the change in the linear trend from the control period to the intervention period. Parameter estimates were calculated, along with odds ratios, 95% confidence intervals and corresponding p-values.

Site specific models

Models were prepared for each individual hospital using a Generalized Linear Model (GLM) assuming a Binomial distribution with logit link. The linear predictor was:

$$\text{log}\left( \frac{p_{t}}{1-p_{t}} \right)=\beta_{0}+\beta_{1}t+\beta_{2}inter_{t}+\beta_{3}t\cdot inter_{t}$$

where $p_{t}$was the proportion of patients reporting at time $t$ ($t$ centred on the first site visit for each hospital so that time was negative during the control period, was equal to 0 at the time of the first site visit, and was positive during the intervention period), $inter_{t}$is an indicator variable taking on the value 0 before the first site visit and the value 1 at and after the first site visit, the term $t\cdot inter_{t}$ is an interaction variable between time and the intervention indicator to allow for different slopes in the control and intervention periods.

To account for additional variance in some of the site-specific models, a dispersion parameter was included when appropriate (making it quasi-Binomial). Overdispersion was accounted for by this parameter if the sum of the squared deviance residuals divided by the residual degrees of freedom was greater than 1.5. Models in which a dispersion parameter was used are identified as over dispersed in the relevant Tables in the paper.

Combined hospital models

To model all sites simultaneously and estimate the average intervention effect, a GLMM was estimated assuming a Binomial distribution with logit link. A random intercept, slope and intervention effect (on both intercept and slope) was assumed with respect to each hospital. The linear predictor had the form:

$$\log\left( \frac{p_{it}}{1-p_{it}} \right)=\beta_{0}+\gamma_{i0}+\left( \beta_{1}+\gamma_{i1} \right)t+\left( \beta_{2}+\gamma_{i2} \right)inter_{it}+\left( \beta_{3}+\gamma_{i3} \right)t\cdot inter_{it}$$

Where $p_{it}$ was the proportion of patients in which the outcome occurred for hospital $i$ at time $t$ and the covariates are as defined above, except now they vary with respect to the hospital index, $i$. The $\gamma_{ij}$ term gives the deviation from the mean value of the $j$th coefficient for the $i$th hospital; these are the random effects. The random effects are assumed to be correlated and from a Normal distribution with mean 0, i.e. $\gamma\sim N(0,\Sigma)$.

For the incomplete ED visit outcome there were issues with the convergence of the combined model when using the logit link due to data instability, attributed to fewer years of data for hospital 8. For this outcome, the complementary log-log link was used instead of the logit link, as this approach better handles skewed and converged data. The complementary log-log link linear predictor had the following form:

$$\log\left( -log\left( 1-p_{it} \right) \right)=\beta_{0}+\gamma_{i0}+\left( \beta_{1}+\gamma_{i1} \right)t+\left( \beta_{2}+\gamma_{i2} \right){inter}_{it}+\left( \beta_{3}+\gamma_{i3} \right)t\cdot{inter\text{ }}_{it}$$

Intervention effects

The GLMM approach allows estimation of the intervention effect at an average hospital (referred to as the population level prediction) as well as at each individual hospital considered in the study. The population level predictions were conditional (random effects set to zero) as opposed to marginal (integration over the random effects); the interpretations of each type of prediction differ and the conditional predictions were deemed more appropriate for this scenario. The predictions differ because the population-average evolution (marginal prediction) is not the same as the evolution for an average hospital (conditional prediction with random effects all set to zero). Given that implementation occurs at an individual hospital the conditional predictions were considered more informative for the intervention effect if implemented at a new hospital.

The parameters of most interest in all models are:

$\beta_{1}$, the control period linear trend (pre-intervention slope) in terms of the log-odds (or complementary log-log for the incomplete ED visit outcome) of the outcome occurring.

$\beta_{3}$, the change in the linear trend (change in slope) from the control period to the intervention period in terms of the log-odds of the outcome occurring.

$\beta_{1}+\beta_{3}$, the intervention period linear trend (intervention slope) in terms of the log-odds of the outcome occurring (the combined pre-intervention trend, and the change in trend associated with the intervention).

The contrasts of interest listed above were exponentiated when appropriate to derive odds ratios which were presented along with 95% confidence intervals (CI). P-values for fixed effects are based on Wald tests using z-scores; likelihood ratio tests gave similar values.

Data management and aggregations were performed in SAS version 9.3. The analyses were conducted in R version 3.2.1 (2015-06-18) using the glm and glmer functions from the stats and lme4 (version 1.1.8) packages respectively (Bates, Maechler, Bolker and Walker 2015). In the case of GLMMs using glmer, the estimation method used was the BOBYQA algorithm (Powell 2009). Figures were constructed using the ggplot2 (Wickham 2009, version 1.0.1) package.

***References***

Bates D, Maechler M, Bolker B and Walker S (2015) lme4: Linear mixed-effects models using Eigen and S4. R package version 1.1-8, <URL: <http://CRAN.R-project.org/package=lme4>>.

Powell MJD (2009), "The BOBYQA algorithm for bound constrained optimization without derivatives", Report No. DAMTP 2009/NA06, Centre for Mathematical Sciences, University of Cambridge, UK. <http://www.damtp.cam.ac.uk/user/na/NA_papers/NA2009_06.pdf>.

Wickham H, ggplot2: elegant graphics for data analysis. Springer New York, 2009.
