# Supplementary material for: Can a continuous quality improvement program create culturally safe emergency departments for Aboriginal people in Australia? A multiple baseline study
Source: BMC Health Serv Res. 2019 Apr 11;19:222. doi: 10.1186/s12913-019-4049-6 (PMC6458761; doi:10.1186/s12913-019-4049-6)
Supplement: Supplementary file 2 — Factors influencing implementation. Description of data: A detailed description of the factors influencing implementation, as reported by key informant interviewees. (DOCX 19 kb) [file 12913_2019_4049_MOESM2_ESM.docx]

**ADDITIONAL FILE 2**

***Factors influencing implementation***

Following implementation of the Aboriginal Identification in Hospitals Quality Improvement Program (AIHQIP), qualitative interviews of 15-60 minutes duration were conducted with participating Emergency Department (ED) staff and other hospital staff (n=23) exploring factors influencing project implementation and perceived achievements. Interviewees’ views on the factors influencing implementation are described below:

Supportive policy context

It was observed that the AIHQIP aims aligned with some state-wide strategies and policies relating to Aboriginal health and the identification of Aboriginal patients in hospitals. It was felt that this alignment led to senior hospital staff actively supporting the delivery and governance of continuous quality improvement (CQI) projects. This support helped project officers to engage staff in project processes:

*“The AIHQIP was consistent with the Local Health District and state policy initiatives regarding Aboriginal identification and so this really helped me get buy in from the Local Health District and hospital executives”.* (Local Health District Aboriginal staff member, Hospital 1)

Leadership from senior hospital staff

It was felt that hospitals with committed ‘champions’ in hospital executives, senior ED managers, and quality staff were most likely to implement the nine-step CQI framework thoroughly and to achieve sustainable organisational outcomes. Conversely, inadequate input from senior staff was identified as an implementation barrier. The establishment of project working groups was identified as an effective strategy in ensuring hospital executive buy in to local CQI projects:

*“The General Manager was right behind the project with a high level of engagement and he kept actively following up the implementation with senior staff and he asked for reports at executive meetings. This was essential to the success of our project”.* (Social Worker Manager, Hospital 7)

Aboriginal advisory structures

It was felt that having formal Aboriginal advisory structures for projects enabled Aboriginal community representatives to share their ideas and suggestions for improving the provision of care to Aboriginal patients with hospital staff, including senior managers. In hospitals in which these formal structures already existed, the project officer was able to quickly and effectively build connections and consult with local Aboriginal community-controlled organisations.

Project working groups

It was reported that the establishment of project working groups was beneficial in engaging stakeholders in project processes. It was felt that certain staff strengthened the functioning of project working groups, including:

- ED staff with responsibility for managing and/or reporting ED patient information. These staff enabled the use of data to inform project processes.
- Staff from the hospital quality unit or the ED quality officer. These staff supported the adoption of project strategies in other hospital wards and sites.
- Aboriginal health staff. These staff increased the working group’s capacity to engage with Aboriginal patients and other Aboriginal health staff.

Building understanding and shared meaning

Building understanding and shared meaning between project working group members and the broader ED team was considered essential to achieving practice and systems changes in EDs. The following activities were identified as helping to build shared meaning across project stakeholders: provision of cultural awareness training for ED staff; feedback to ED staff on findings and outcomes of project consultations with Aboriginal people; the establishment of links between ED workers and staff in Aboriginal community-controlled organisations; and informal discussions between hospital-based Aboriginal staff and ED staff:

*“...the project built relationships between the Aboriginal and non-Aboriginal staff and they then understood how important the follow up was. This type of communication about these issues was not there before the project – an unexpected positive outcome”*. (Director Allied Health, Hospital 8)

Relationships between Aboriginal health staff and the ED team

It was felt that most hospitals had success in increasing ED staff’s understanding of the roles of Aboriginal Liaison Officers (ALOs) and other Aboriginal health staff and in engaging these staff in project processes. It was felt that this resulted in increased referrals to hospital ALOs and improved the cultural safety of EDs for some Aboriginal patients, although these were not directly measured:

*“Some ED staff certainly have a new understanding of the importance of culture and the role of Aboriginal staff in providing a good service to Aboriginal patients and they are more confident to speak with Aboriginal patients and staff...they have a higher rate of referral to and use of those Aboriginal staff than before the project”.* (Nurse Manager, Hospital 3)

Tailored cultural awareness training

It was considered that implementing tailored cultural awareness training in some hospitals increased some ED staff’s understanding of Aboriginal culture and the needs of Aboriginal patients, and led to some staff reflecting on and altering their practice accordingly. It was also reported that such training assisted ED staff to improve collaboration with Aboriginal health staff.

Engaging Aboriginal organisations and communities

Early positive engagement of Aboriginal community-controlled organisations and local Aboriginal community members in project processes was identified as a key enabling factor:

*“The Aboriginal people on the working group were committed, passionate and engaged. They were so keen to make the future better for their children. They gave lots of positive input on project priorities, strategies and gave us great links into the communities”.* (Project Officer, Hospital 1).

For some participating hospitals, however, the development of partnerships with Aboriginal community-controlled organisations proved challenging.

Aboriginal Liaison Officers

It was felt that the participation of hospital ALOs in project processes was essential. These staff assisted project working groups by providing insights into the experiences of Aboriginal patients and by facilitating links with local Aboriginal communities. Experienced ALOs who were knowledgeable about Aboriginal patients, local Aboriginal communities and ED and hospital processes were considered particularly helpful:

*“The key thing was the ALO. Without her I couldn’t have done it. She really knew her local community and she linked me in and I was able to build trust because of her”.* (Project Officer, Hospital 6)

Project officers

The employment of dedicated project officers was considered crucial to successful implementation of local CQI projects, given the busy nature of ED settings. It was reported that certain characteristics in project officers facilitated project success, especially strong project management skills, a good understanding of hospital systems, and an ability to effectively communicate and build relationships with local Aboriginal communities. It was also felt that Aboriginal project officers were particularly successful in engaging with local Aboriginal communities:

*“Dedicated funds that enabled us to employ an excellent project officer were necessary. She was culturally sensitive and aware, had good management skills and understood the ED pressures”*. (Social Worker Manager, Hospital 7)

The nine-step CQI framework and associated training and support

It was reported that the nine-step CQI framework used in the AIHQIP provided project working groups with a structured and clear set of steps for implementing local projects in a culturally competent way. Formal training in implementing the nine-step CQI framework was also considered essential to the success of projects. Site visits from the AIHQIP team also supported the implementation of local projects, especially in sites in which key implementation staff were inexperienced in CQI processes and/or working with Aboriginal people:

*“The training and support were essential. She (AIHQIP staff) was fantastic, so supportive, knowledgeable and clear about the process and she was always available when I needed her. The information and Toolkit were great too”.* (Primary, Community Allied Health Manager, Hospital 1)
